# Remitting Seronegative Symmetrical Synovitis with Pitting Edema Syndrome in a Chronic Hemodialysis Patient

**DOI:** 10.1155/2012/371795

**Published:** 2012-01-29

**Authors:** Shunsuke Yamada, Seiya Fuyuno, Masahiro Eriguchi, Kazuhiko Tsuruya, Takanari Kitazono

**Affiliations:** ^1^Department of Medicine and Clinical Science, Graduate School of Medical Sciences, Kyushu University, Fukuoka 812-8582, Japan; ^2^Department of Integrated Therapy for Chronic Kidney Disease, Graduate School of Medical Sciences, Kyushu University, Fukuoka 812-8582, Japan

## Abstract

A 75-year-old male who was undergoing chronic hemodialysis developed abrupt-onset pitting edema and pain in the dorsum of both hands and feet. Biochemical analysis disclosed increased C-reactive protein, and negative rheumatoid factor and antinuclear antibody. Radiological examination showed no bony erosion. Computed tomography and gallium scintigraphy revealed no active infection or neoplasms. The clinical diagnosis was remitting seronegative symmetrical synovitis with pitting edema (RS_3_PE) syndrome. The pitting edema and inflammatory response quickly subsided after low-dose prednisolone therapy. This case demonstrates that RS_3_PE syndrome could be a differential diagnosis in elderly patients undergoing dialysis who develop pitting edema and joint pain.

## 1. Introduction

Remitting seronegative symmetrical synovitis with pitting edema (RS_3_PE) syndrome is a distinct disease entity that was first described by McCarty et al. in 1985 [1]. RS_3_PE syndrome is characterized by symmetrical and acute synovitis, marked pitting edema, the absence of rheumatoid arthritis, increased acute phase reactants, lack of bony erosions on radiography, and a benign and short clinical course, as evidenced by quick responsiveness to low-dose prednisolone treatment and good prognosis after tapering oral prednisolone [2]. Although dialysis patients often experience articular pain related to amyloidosis and other musculoskeletal disorders [3], RS_3_PE syndrome has rarely been reported.

We describe here the case of a hemodialysis patient who developed acute-onset pitting edema in the dorsum of the bilateral hands and feet. He was finally diagnosed with RS_3_PE syndrome and was successfully treated with low-dose oral prednisolone.

## 2. Case Report

A 75-year-old male with a 23-year history of chronic hemodialysis for end-stage renal failure developed acute-onset bilateral wrist pain, accompanied by dorsal edema of the bilateral hands and feet, increased acute phase reactants, and low-grade fever. These symptoms started 3 months before admission. His past medical history included coronary artery bypass grafting and aortic valve replacement 3 years prior to admission. The pitting edema was initially attributed to extracellular fluid overload, but persisted after the volume status was adjusted.

**Figure 1:**
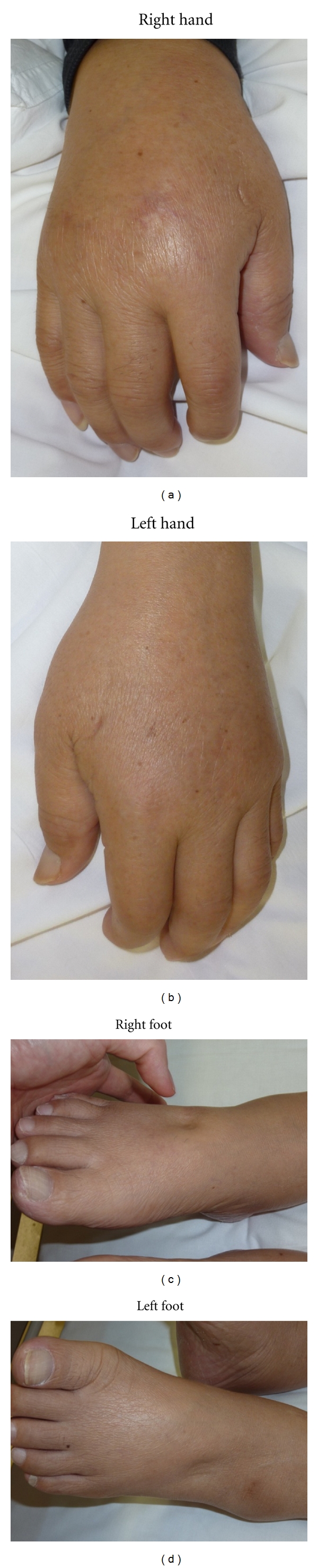
Photographs of the bilateral hands and feet. Edematous hands and feet with pitting edema were observed.

On admission, the patient was alert, his blood pressure was 108/60 mmHg, heart rate was 64 beats per minute, and body temperature was 36.8°C. Physical examination revealed bilateral swelling of the hands and feet with pitting edema (Figure 1). He also showed minimal tenderness and tenosynovial thickening at the wrist, and metacarpophalangeal and proximal interphalangeal joint tenderness. Laboratory test results disclosed serum levels of C-reactive protein of 12.8 mg/dL, hemoglobin of 8.5 g/dL, white blood cell count of 8400/*μ*L with neutrophils 78%, albumin 2.4 g/dL, and total protein 5.6 g/dL. Rheumatoid factor, antinuclear antibody and anti-Scl-70 antibody were all negative. The results of *γ*-interferon-release assay, repeated blood cultures, and serum procalcitonin test were negative. Plain X-radiography showed no joint erosion in either hand. Enhanced computed tomography showed no signs of neoplasm or active infection. No accumulation in the juxta-articular regions was found on gallium scintigraphy. After active infection and neoplasm were ruled out, the patient was finally diagnosed with RS_3_PE syndrome [1, 2].

Twenty mg/day of oral prednisolone was initiated on the 12th hospital day, after which the wrist pain and pitting edema immediately subsided, followed by a decrease in serum C-reactive protein levels to 0.3 mg/dL on the 28th hospital day. At 6 months after discharge, the prednisolone dose was gradually tapered to 5 mg/day, and the patient remained free from pitting edema and joint pain.

## 3. Discussion

The etiology of RS_3_PE syndrome remains unclear. However, recent clinical studies have clarified some aspects of its pathogenesis. Cantini et al. revealed that tenosynovitis of both extensor and flexor tendons were involved in the development of edema of the subcutaneous and peritendinous soft tissue, and that the predominance of extensor tenosynovitis at the wrist and feet is a hallmark of RS_3_PE syndrome [4]. Arima et al. recently showed that vascular endothelial growth factor played a role in the pathological changes responsible for both hypervascularity (synovitis) and vascular permeability (subcutaneous pitting edema) [5]. In addition, possible associations with HLA B7 and HLA A2 [6] neoplasms and infectious diseases [7] have been identified, indicating the involvement of autoimmune processes in the pathogenesis of RS_3_PE syndrome.

Recent advances in imaging technology have contributed to the accurate diagnosis of RS_3_PE syndrome, because important pathological findings such as subcutaneous edema and tissue thickening, tenosynovitis, subcutaneous fluid collection, dilatation of lymphatic vessels, and increased blood flow, cannot be fully detected by physical examination [4, 8]. Ultrasonography and magnetic resonance imaging can sometimes detect these disease-related changes, but both these imaging modalities are associated with advantages and disadvantages [8]. Thus, the appropriate and combined use of physical examination and imaging techniques should be used to aid the early diagnosis of RS_3_PE syndrome.

A clinical diagnosis of RS_3_PE syndrome should be made with care, because the clinical presentation of several disease entities, such as polymyalgia rheumatica (PMR), late-onset rheumatoid arthritis, and other rheumatic disorders (spondyloarthropathies, psoriatic arthritis, and sarcoidosis) can occasionally mimic that of RS_3_PE syndrome [9]. PMR is an important disease entity that affects the elderly, and also manifests as increased serum inflammatory markers, onset in older age, proximal muscle pain, and rapid response to low-dose prednisolone. Differentiation between PMR and RS_3_PE syndrome may be difficult, but could be confirmed by the extension of the synovitis and the presence of distal pitting edema, the predominance of peripheral arthritis, a good, rapid responsiveness to low-dose oral prednisolone without relapse, and lack of concomitant neoplasm. However, Salvarani et al, in a retrospective cohort study of 245 patients with PMR, reported a prevalence of extremity swelling with pitting edema in 19 (8%) of patients [10]. They concluded that the distal swelling with pitting edema in patients were peripheral manifestations of the inflammatory process associated with PMR, most likely due to vigorous tenosynovitis. Actually, some clinicians have recently insisted that RS_3_PE syndrome may be a feature of different diseases, and not a distinct disease entity [11]. Further clinical examinations are thus required to determine whether or not RS_3_PE syndrome is a distinct disease, or merely a clinical feature of different inflammatory diseases.

Finally, RS_3_PE syndrome has not previously been reported in dialysis patients. This is probably because RS_3_PE syndrome is a relatively rare disease that is not well known in the nephrology community. In addition, dialysis patients can easily be underdiagnosed, because acute-onset wrist pain with distal pitting edema might be attributed to simple accumulation of extracellular fluid and arthritis related to amyloidosis or chronic inflammation, which are often seen in patients on long-term dialysis. The present case report thus emphasizes the importance of considering RS_3_PE syndrome as a potential differential diagnosis in dialysis patients presenting with acute arthritis with pitting edema in the distal extremities.

In conclusion, we report here a case of a chronic hemodialysis patient who developed RS_3_PE syndrome. This case suggests that RS_3_PE syndrome should be considered in the differential diagnosis of polyarthritis in dialysis patients who present with abrupt-onset synovitis and pitting edema in the dorsum of the bilateral extremities, fever, and increased inflammatory response.
